# Pan-genome analysis identifies a *Burkholderia cepacia*-specific gene marker for rapid species-level identification

**DOI:** 10.3389/fmicb.2026.1915686

**Published:** 2026-09-23

**Authors:** Kun Cheng, Shuaicheng Mu, Huifen Feng, Rui Liu, Jie Liu, Ling Li, Jiaqi Liu, Weiwei Wu, Yuanyuan Ge, Lei Zhai, Su Yao

**Affiliations:** 1 China Center of Industrial Culture Collection, China National Research Institute of Food and Fermentation Industries CO., LTD., Beijing, China; 2 China National Accreditation Service for Conformity Assessment, Beijing, China

**Keywords:** *Burkholderia cepacia*, gene marker, pan-genome, PCR, species-level identification

## Abstract

**Inroduction:**

*Burkholderia cepacia* is a representative member of the *Burkholderia cepacia* complex (Bcc) and is widely distributed in water and soil environments. It has a propensity to form biofilms in water-based products, making it a significant contaminant in the pharmaceutical, cosmetic, and food industries and posing threats to product quality control and public health. Therefore, rapid and accurate species-level identification of *B. cepacia* is of great importance.

**Methods:**

To achieve rapid and accurate species-level identification of *B. cepacia*, we performed a pan-genome analysis based on 138 high-quality genome sequences covering *B. cepacia* and its closely related Bcc species with taxonomic positions validated by average nucleotide identity (ANI) analysis. A conserved core gene encoding a glycosyl hydrolase family 18 (GH18) protein was identified as a species-specific molecular marker. A specific primer pair comprising SEQ ID NO.2 and SEQ ID NO.3 was designed based on this marker and validated with both genome sequences and bacterial pure cultures.

**Results:**

The GH18-encoding gene is highly conserved across 115 *B. cepacia* strains (sequence identities ranging from 90.3 to 100.0%) and is absent in all other Bcc species examined. The primer pair exhibited high inclusivity and exclusivity for *B. cepacia*. The established PCR method achieved a detection limit of 5 pg/μL for pure genomic DNA and 10^3^ CFU/mL in artificially contaminated water-based personal care and medical device products.

**Discussion:**

The identification of this conserved core gene provides a promising basis for the rapid and accurate species-level detection of *B. cepacia*. Given the widespread occurrence of *B. cepacia* in water and soil environments and its ability to form biofilms in water-based products, such a species-specific molecular marker may help improve contamination monitoring and quality control in the pharmaceutical, cosmetic, and food industries, thereby contributing to public health protection.

## 1 Introduction

The species *Burkholderia cepacia* was established by Yabuuchi et al. (1992) based on the previously described *Pseudomonas cepacia* (Palleroni and Holmes, 1981), and it currently represents the type species of the genus *Burkholderia*, belonging to the family *Burkholderiaceae*, order *Burkholderiales*, class *Betaproteobacteria*, and phylum *Pseudomonadota*. Strains of *B. cepacia* are Gram-negative, obligately aerobic and rod-shaped bacteria that possess motile flagella (Palleroni and Holmes, 1981; Garrity et al., 2005). They are widely distributed in diverse ecological niches, including rotten onions, soils, various natural materials, and clinical specimens (Garrity et al., 2005). Originally recognized as a plant pathogen (Burkholder, 1950), *B. cepacia* has since been established as both an environmental bacterium and an opportunistic human pathogen. *B. cepacia* is one of the key pathogens isolated from cystic fibrosis (CF) patients, recognized as a serious opportunistic human pathogen for being associated with nosocomial infections (Garrity et al., 2005; Mahenthiralingam et al., 2005; Aytaç et al., 2025). Transmission of *B. cepacia* bacteria to susceptible individuals can occur through person-to-person contact, environmental exposure, or contaminated products (Mahenthiralingam et al., 2005; Tavares et al., 2020). While CF patients are at particularly high risk due to their increased susceptibility to chronic pulmonary colonization, immunocompromised individuals and hospitalized non-CF patients are also vulnerable to *B. cepacia* infections (Mahenthiralingam et al., 2005; Coulon et al., 2021). In addition, contamination of water-based non-sterile pharmaceutical, cosmetic, and personal care products poses a risk to the general population, as these products are used by both CF and non-CF individuals (Tavares et al., 2020; Seelman et al., 2022). Notably, clinical and environmental isolates of the *Burkholderia cepacia* complex (Bcc) have been shown to differ in various phenotypic and genetic traits, including the production of specific secondary metabolites (Coulon et al., 2021). Many isolates of Bcc exhibit significant biofilm-forming capabilities and intrinsic multidrug resistance, leading to considerable contamination risk in water-based pharmaceutical products (Tavares et al., 2020), cosmetics (Seelman et al., 2022; Doran et al., 2025), and food products (Moore et al., 2001). Therefore, accurate species-level identification of *B. cepacia* is crucial for scientific research, industrial applications, and clinical management.

However, *B. cepacia* is not an isolated taxon. It belongs to Bcc, a group of closely related species that share high phenotypic similarity but are genetically distinct. The Bcc currently comprises 24 validly described species, including *B. aenigmatica*, *B. ambifaria*, *B. anthina*, *B. arboris*, *B. catarinensis*, *B. cenocepacia*, *B. cepacia* (the type species, i.e., *B. cepacia sensu stricto*), *B. contaminans*, *B. diffusa*, *B. dolosa*, *B. lata*, *B. latens*, *B. metallica*, *B. multivorans*, *B. orbicola*, *B. pseudomultivorans*, *B. puraquae*, *B. pyrrocinia*, *B. seminalis*, *B. stabilis*, *B. stagnalis*, *B. territorii*, *B. ubonensis* and *B. vietnamiensis* (Jin et al., 2020). Although some physiological traits, such as the ability to utilize certain carbohydrates (e.g., D-arabinose), have been reported to aid in differentiating *B. cepacia* from other Bcc species (Garrity et al., 2005), these phenotypic characteristics are often variable and heavily dependent on the culture conditions and operator expertise, rendering them unreliable for routine identification. Distinguishing *B. cepacia sensu stricto* from its close relatives within Bcc therefore remains particularly challenging using conventional manual or automated phenotypic methods, such as selective media, VITEK 2 and Matrix-Assisted Laser Desorption/Ionization Time of Flight Mass Spectrometry (MALDI-TOF MS) (Devanga Ragupathi and Veeraraghavan, 2019; Furlan et al., 2019). Molecular methods targeting the 16S rRNA gene are also insufficient, as its sequence identity among Bcc species ranges from 97.87 to 100% (Jin et al., 2020). While *recA* is a conserved housekeeping gene marker, its effectiveness is limited when discriminating closely related species within Bcc (Jin et al., 2020). Other markers, such as *rpsU*, have been explored but suffer from inadequate interspecies resolution and accuracy (Devanga Ragupathi and Veeraraghavan, 2019). Although more recent techniques like multilocus sequence typing (MLST) and whole-genome sequencing (WGS) offer promising alternatives to differentiate Bcc species, they remain labor-intensive and costly for routine use (Spilker et al., 2009; Jin et al., 2020). Therefore, a rapid, accurate and cost-effective method for species-level identification of *B. cepacia sensu stricto* is urgently needed.

The development of specific primers targeting novel DNA markers identified through whole-genome-based approaches has demonstrated significant potential for the identification of diverse microorganisms such as *Leuconostoc* (Kim et al., 2022), *Enterococcus* (Xiang et al., 2023), *Lacticaseibacillus paracasei* (Guo et al., 2024) and *Pseudomonas aeruginosa* (Quiroz-Morales et al., 2023). Advances in high-throughput sequencing technology have led to public release of vast amounts of microbial genomic data. Pan-genome analysis, which characterizes the core, accessory and unique gene sets of microorganisms, has emerged as a powerful tool for discovering novel gene markers (Kim et al., 2020). Polymerase chain reaction (PCR)-based methods targeting such specific gene marker offer notable advantages over alternative identification techniques, including rapidity, high sensitivity, and operational convenience, thereby providing a user-friendly and cost-effective solution for bacterial detection in practical applications.

In this study, a species-specific molecular marker for *B. cepacia sensu stricto* was identified using pan-genome analysis of 115 *B. cepacia* genomes and 23 type strains representing all other Bcc species. Four primer pairs targeting this marker were designed and subjected to *in silico* specificity assessment. Following experimental validation of amplification efficiency, inclusivity, and exclusivity, one highly robust primer pair was selected. This primer set enables the rapid and accurate detection of *B. cepacia*.

## 2 Materials and methods

### 2.1 Genome sequences

We retrieved a total of 283 *B. cepacia* genome sequences available in the National Center for Biotechnology Information (NCBI) database at the retrieval date (April 18, 2024), along with the genome sequences of the type strains of 23 close related species within Bcc: *B. aenigmatica*, *B. ambifaria*, *B. anthina*, *B. arboris*, *B. catarinensis*, *B. cenocepacia*, *B. contaminans*, *B. diffusa*, *B. dolosa*, *B. lata*, *B. latens*, *B. metallica*, *B. multivorans*, *B. orbicola*, *B. pseudomultivorans*, *B. puraquae*, *B. pyrrocinia*, *B. seminalis*, *B. stabilis*, *B. stagnalis*, *B. territorii*, *B. ubonensis* and *B. vietnamiensis*. All the genome sequences underwent rigorous quality control based on the following criteria: (1) contig count less than 1,000 (Riesco and Trujillo, 2024), (2) total assembly length within ±20% of the expected genome size for the respective species (European Food Safety Authority, 2024), (3) genome completeness >90% (European Food Safety Authority, 2024; Riesco and Trujillo, 2024), and (4) contamination level <5% (European Food Safety Authority, 2024; Riesco and Trujillo, 2024), evaluated with CheckM v1.0.12 (Parks et al., 2015). After quality filtering, 283 raw genome sequences were reduced to 115 high-quality *B. cepacia* genomes, which together with 23 type strains of the closely related Bcc species constituted a final set of 138 high-quality whole-genome sequences, as detailed in Supplementary Table S1. These genome sequences were used in subsequent pan-genome analysis for the development of species-specific primers.

### 2.2 Pan-genome and phylogenetic analysis

To achieve precise genomic classification, the average nucleotide identity (ANI) was calculated using OrthoANI (Lee et al., 2016), with genomes assigned to the same species above the ANI similarity threshold of 96% (Richter and Rosselló-Móra, 2009; Riesco and Trujillo, 2024). The genome annotations were generated for the assembled genomes using Glimmer v3.02 (Delcher et al., 2007) in three sequential steps: (i) long-orfs was run with parameters -n -t 1.15 to identify open reading frames (ORFs); (ii) build-icm was run with default parameters to construct an interpolated context model (ICM); and (iii) the main Glimmer v3.02 program was used to predict genes in the genome based on the ICM model. Following gene prediction, a perl script was used to append genome-specific identifiers to each predicted gene protein sequence, and all protein sequences were concatenated into a single gene file for subsequent analysis. Core-genome and pan-genome phylogenetic analyses were conducted with CD-HIT v4.6 (Fu et al., 2012) and TreeBeST v1.9.2 with parameters nj and -b 1,000 (Vilella et al., 2009). And the 23 type strains of the other Bcc species were used as the outgroup for rooting the phylogenetic tree.

Orthologous gene clusters were generated using CD-HIT v4.6 with a ≥50% sequence identity threshold and ≥70% coverage threshold (-c 0.5 -n 3 -p 1 -g 1 -s 0.7 -aL 0.7 -aS 0.7). This relatively permissive threshold was chosen to maximize the capture of candidate genes in the initial screening phase, following previously established practices for gene clustering in bacterial pan-genome studies (Addou et al., 2009). The species-specificity of the candidate genes identified through this initial clustering was subsequently validated through multiple independent approaches, including BLASTn searches against the NCBI core_nt database (as described in Section 2.3) and re-analysis using Roary v3.13.0 with a stringent 95% identity threshold, which confirmed the species-specific distribution of the selected marker (Supplementary Table S2).

Based on their prevalence across the 138 Bcc genomes, the clustered genes were classified into four categories: core genes (present in 100% of the genomes), soft-core genes (~95 to 100%), accessory genes (~15 to 95%), and unique genes (~0 to 15%) (Kim et al., 2020). Core-genome and pan-genome phylogenetic analyses were subsequently performed. For core gene sequence alignment, multiple sequence alignment was performed with MUSCLE v3.8.1551 with default parameters. Phylogenetic trees based on both core gene alignments and the pan-genome presence/absence matrix (derived from ≥50% identity and 70% coverage threshold) were reconstructed and visualized using iTOL v7.2.1 (Letunic and Bork, 2024).

### 2.3 Screening species-specific genes and design of primers

To identify species-specific genes, we performed an analysis following the workflow outlined below. The pan-genome gene set generated in Section 2.2 was summarized in the form of a binary (0/1) matrix representing gene presence/absence by a perl script and then visualized by R base v4.3.2. Candidate single copy species-specific genes for *B. cepacia* were identified by applying a strict criterion: complete presence in the target genome and complete absence in all non-target genomes. The specificity of these candidate sequences in the *B. cepacia* species was further examined through BLASTn searches against the NCBI core_nt database (retrieved on July 15, 2024) using default parameters, and unqualified candidate sequences were filtered out. To obtain functional annotation, the predicted protein sequences of the final candidate genes were compared against the UniProt database (UniProtKB 2025_01) using BLASTp. Functional information was assigned based on the annotated homologous proteins identified in UniProt. Finally, primers targeting the selected *B. cepacia* species-specific genes were designed by using Primer Premier v6.0 software (Singh et al., 1998). The parameters for primer design were set using the default parameters in Primer Premier v6.0: melting temperature (Tm) 50.0 ± 5 °C, primer length 18–22 bp, GC content 40–60 mol%, and avoidance of secondary structures. The amplicon length was set as 250–700 bp. The candidate primer pairs were shown in Table 1, along with their calculated Tm values. The oligos were synthesized and purified by PAEG from Sangon Biotech Co., Ltd. (Shanghai, China).

**Table 1:** Four candidate *B. cepacia*-specific primer pairs designed in this study.

Target gene/Function	Primer name	Primer sequence (5′–3′)	Position in SEQ ID NO.1 (5′–3′)	Length (bp)	Tm (°C)	Size of amplicon (bp)	GC content (mol%)	Note
SEQ ID NO.1/Glycosyl hydrolase family 18 protein (accession number: GCA_003546465.1, 3712308-3713663)	SEQ ID NO.2	CTACGGTCAATAGCCAGTT	572–590	19	49.7	529	47.4	Final primers
	SEQ ID NO.3	GGATCGTCAAGCGGAATA	1,083–1,100	18	49.4		50.0	
	SEQ ID NO.4	CTTGAACGAAGACGAACC	39–56	18	48.8	457	50.0	–
	SEQ ID NO.5	GCGAATTGCAGAGAACAGAA	476–495	20	49.7		40.0	
	SEQ ID NO.6	TTGCGAATAATCTGGTGACA	371–390	20	50.7	300	40.0	–
	SEQ ID NO.7	CAATCCTGGACGGCGATA	653–670	18	51.6		55.6	
	SEQ ID NO.8	GGTTCCACGATCCAGATC	175–192	18	49.6	410	55.6	–
	SEQ ID NO.9	CTATTGACCGTAGCGTAATC	565–584	20	49.3		45.0	

### 2.4 Bacterial strains and DNA extraction

A total of 68 reference strains were utilized in this study, sourced from the China Center of Industrial Culture Collection (CICC) (Supplementary Table S3). Each strain was cultured aerobically on Tryptic Soy Agar (TSA) plates at 37 °C for 24 h. Bacterial cells were harvested via centrifugation at 12,000×*g* for 5 min, and genomic DNA was extracted using the E.Z.N.A. Bacterial DNA Kit (Omega Bio-Tek, GA) following the manufacturer’s protocols. The purity and concentration of the extracted DNA was assessed using a Biodrop μLite+ spectrophotometer (BioDrop, USA). DNA samples with concentration greater than 50 ng/μL and *A*_260/280_ ratios within 1.8–2.0 were stored at −20 °C for subsequent use in PCR amplification.

### 2.5 PCR assay

Each PCR reaction was performed in a final volume of 50 μL, including 25.0 μL of 2× EasyTaq PCR SuperMix (TransGen Biotech, Beijing, China), 2.0 μL DNA template, 1.0 μL of forward primer (10 μM), 1.0 μL of reverse primer (10 μM), and sterilized ddH_2_O added up to 50 μL. Reactions without the DNA template were set as blank control. The PCR amplification was performed using VeritiPro thermal cycler (ThermoFisher, Singapore). The PCR thermal profile consisted of an initial incubation at 95 °C for 5 min, followed by 35 cycles of denaturation at 95 °C for 30 s, annealing at 55 °C for 30 s, and extension at 72 °C for 30 s, with a final extension at 72 °C for 10 min. The annealing temperature was optimized through gradient PCR over a range of 47–59 °C (in 2 °C increments) and finalized at 55 °C (Supplementary Figure S1). After amplification, PCR products were electrophoresed on 1.0% agarose gel stained with GoldView I nucleic acid staining reagent (10,000×) (Solarbio, Beijing, China) in 1 × TAE buffer. The gel was run at a voltage of 100 V for 20 min and subsequently imaged on the Gel Doc EZ imaging system (Bio-Rad, USA).

### 2.6 Specificity evaluation of species-specific primers using *in silico* and *in vitro* analyses

To assess the specificity of the developed species-specific primers (Table 1) for *B. cepacia* identification, we performed both *in silico* and experimental evaluations. First, an *in silico* PCR analysis was conducted to examine the inclusivity and exclusivity of the primer sequences against a reference database comprising 283 genome sequences of target strains and 1,829 genome sequences of non-target strains retrieved from GenBank (Supplementary Table S4). Genome sequence fragments shorter than 1,000 bp were excluded from subsequent analysis. Primer-template matching was evaluated using the Hamming distance implemented in the Python-Levenshtein package. Both strands of each genome were searched for correctly oriented primer-binding sites. A maximum of three nucleotide mismatches was allowed per primer, with the exception that mismatches at the terminal 3′ nucleotide were not permitted, as this position is critical for primer extension. The maximum predicted amplicon length was set to 700 bp. Subsequently, the specificity of the primers was experimentally verified using genomic DNA from bacterial pure cultures (Supplementary Table S3). For inclusivity testing, six *B. cepacia* strains were selected: CICC 21624^T^, CICC 10857, CICC 20700, CICC 23648, CICC 24956 and YC15. For exclusivity testing, 62 non-*B. cepacia* strains were used, including 12 strains from other Bcc species and 50 strains from diverse bacterial genera (e.g., *Pseudomonas*, *Bacillus*, *Staphylococcus*, *Escherichia*, and *Acinetobacter*), all of which were expected to yield negative amplification. Genomic DNA extracted from all the tested strains served as the templates for PCR assay.

### 2.7 Detection of *B. cepacia* in spiked water-based matrices

Five types of water-based personal care and medical device products were selected and purchased from online stores, including facial essence, hand sanitizer, makeup remover, mouthwash and sterile saline solution (Supplementary Table S5). All the samples were previously tested and confirmed free of detectable *B. cepacia* by the PCR assay established in this study, and were used for subsequent spiking experiments. The reference strain *B. cepacia* CICC 21624^T^ was cultured and serially diluted 10-fold with sterile normal saline to obtain 2× working suspensions, with theoretical concentrations ranging from 10^8^ to 10^1^ CFU/mL. The actual bacterial concentration in each dilution was verified by the plate counting method. For each spiking simulation, 1 mL of each sample was mixed with 1 mL of the corresponding 2× bacterial suspension, yielding final concentrations of 10^8^ to 10^1^ CFU/mL in a total volume of 2 mL. Uninoculated samples (mixed with 1 mL sterile saline instead of bacterial suspension) were used as negative controls. After thorough vortex mixing, the samples were held static for 10 min, and were subsequently centrifuged at 13,000 rpm for 30 min at 4 °C to pellet the bacterial cells.

To determine the limit of detection (LOD) in different matrices, two independent spiking experiments were performed on separate days. In each experiment, for each matrix, three independent biological replicates were prepared for each spiked concentration of *B. cepacia* CICC 21624^T^. For each biological replicate, independent DNA extraction was performed following the procedure described in section “Bacterial strains and DNA extraction”. Each extracted DNA sample was then subjected to PCR amplification as described in section “PCR Assay”. The LOD was defined as the lowest spiked concentration at which the expected amplicon was consistently detected in all three biological replicates within a single experiment, and confirmed in both independent experiments (i.e., 100% detection rate across all replicates and experiments). To evaluate potential PCR inhibition in the extracted DNA samples, inhibition controls were performed for all samples that yielded negative amplification results. Specifically, 1 μL of purified genomic DNA from *B. cepacia* CICC 21624^T^ was spiked into each negative PCR reaction as an internal amplification control. Successful amplification of the expected band in these control reactions confirmed the absence of PCR inhibitors in the sample DNA extracts.

## 3 Results

### 3.1 Phylogenetic analysis

A total of 138 Bcc genomes were retrieved after quality control, including 115 *B. cepacia* genomes and 23 strains of other Bcc species (Supplementary Table S1). Using these genome sequences, a phylogenetic tree was reconstructed based on the concatenated core gene alignments (Figure 1) to show their relationships. All the 24 Bcc species were clearly separated according to their species names, and the 115 *B. cepacia* strains formed a compact and monophyletic group, suggesting their species taxonomic positions were correct. The topological structure of this phylogenetic tree is generally consistent with previous studies (Jin et al., 2020), highlighting the utility of core-genome phylogeny for classifying closely related Bcc species.

**Figure 1:**
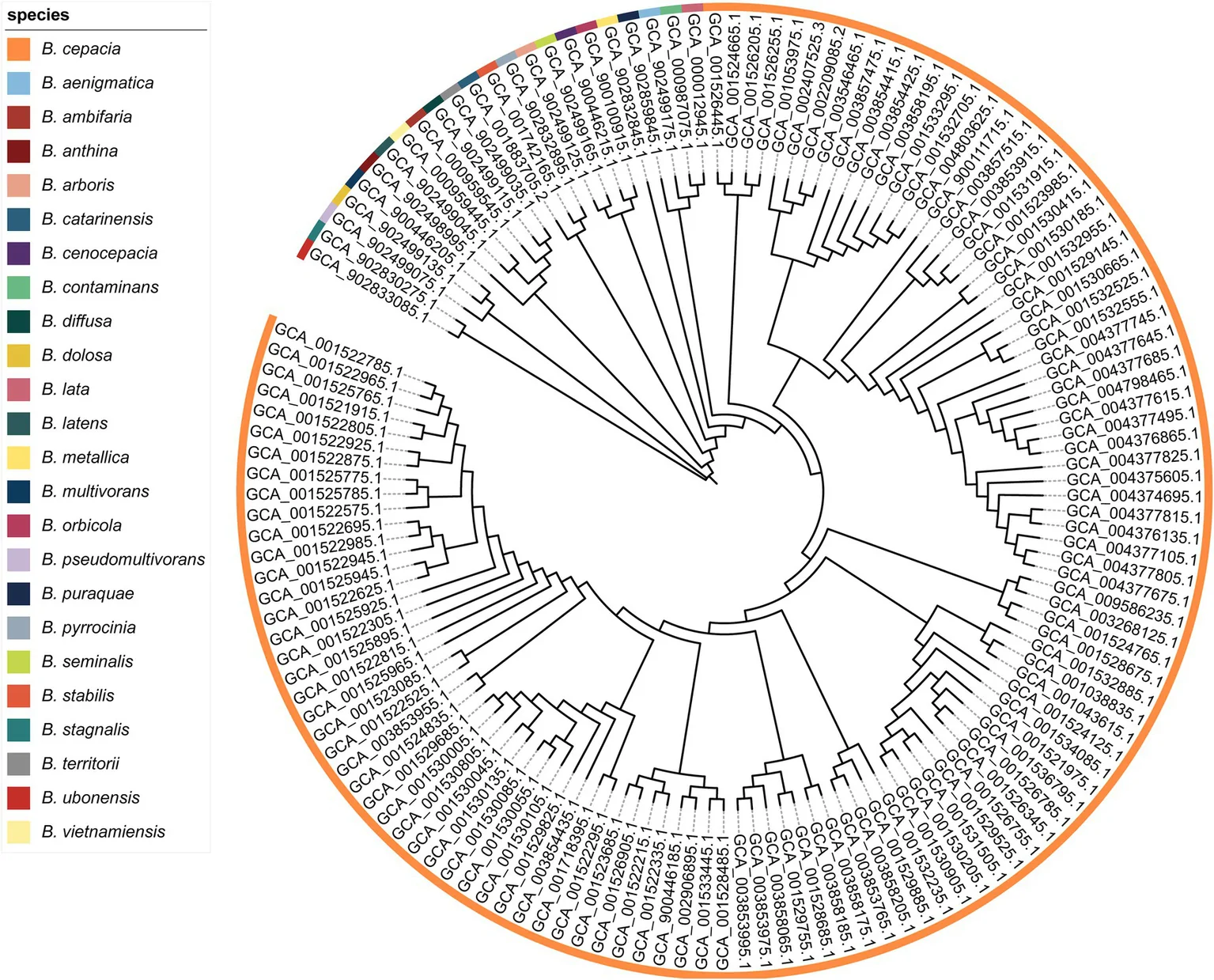
Relationships between *B. cepacia* and other Bcc member species. Pan-genome phylogenetic tree was constructed with concatenated core gene alignments of 115 *B. cepacia* strains and 23 type strains of other Bcc species. Branch lengths were ignored during tree visualization. Therefore, the branch lengths do not represent evolutionary distances.

Average nucleotide identity (ANI) values of genes shared by two genome sequences have become a golden standard for the demarcation of bacterial species, and the ANI values of 95–96% provide a pragmatic tool for bacterial species affiliations in recent years (Richter and Rosselló-Móra, 2009; Riesco and Trujillo, 2024). To further confirm the taxonomic position of the *B. cepacia* strains used for analysis, ANI values were calculated between any two of the 138 Bcc strains (Supplementary Table S6; Figure S2). It was shown that all the 115 *B. cepacia* strains exhibited 96.27–100.00% identity to the type strain *B. cepacia* ATCC 25416^T^, above the ANI threshold for species differentiation, and less than 94.05% identity with other closely related species, confirming the accuracy of their taxonomic classification. Furthermore, a range of 88.01–95.18% ANI values were observed between any of the type strains of all the 24 Bcc species (Supplementary Figure S2), further indicating that ANI values based on whole-genome data can effectively distinguish between closely related Bcc species. The taxonomy accuracy of reference genome sequences is crucial for the development of microbial species identification methods (Kim et al., 2022). Since the taxonomic classification of all the 138 Bcc genome sequences was confirmed to be correct, they were included in subsequent analysis.

### 3.2 Screening species-specific genes with pan-genome analysis

Species-specific molecular markers were screened according to the workflow shown in Figure 2. A total of 138 Bcc genome sequences were subjected to the pan-genome analysis, with *B. cepacia* represented by 115 strains. With CD-HIT v4.6 and a perl script, we recognized genes present/absent in the whole genome sequence dataset, depicted in Figure 3. The number of identified core genes, soft-core genes, accessory genes and unique genes were 2,008, 1,803, 6,040 and 47,871, respectively. *B. cepacia* species-specific genes were defined as being present in all the 115 *B. cepacia* strains but absent in other 23 Bcc species strains. The pan-genome analysis identified five potential *B. cepacia*-specific genes that met this strict criterion. The five candidate genes were further evaluated by BLASTn analysis against 117,743,418 sequence entries in the core nucleotide database (core_nt) in NCBI. This screening narrowed the candidates to a 1,356-bp gene, designated SEQ ID NO.1, which encodes a glycosyl hydrolase family 18 (GH18) protein (Figure 3). This gene was identified as a novel molecular marker for *B. cepacia*, and four candidate pairs of amplification primers were initially designed targeting this sequence (Table 1). The remaining four candidate genes were excluded, as they showed significant sequence similarity (≥90% identity over ≥80% coverage) to genes from other *Burkholderia* species or non-*Burkholderia* taxa in the database, indicating that they were not strictly species-specific.

**Figure 2:**
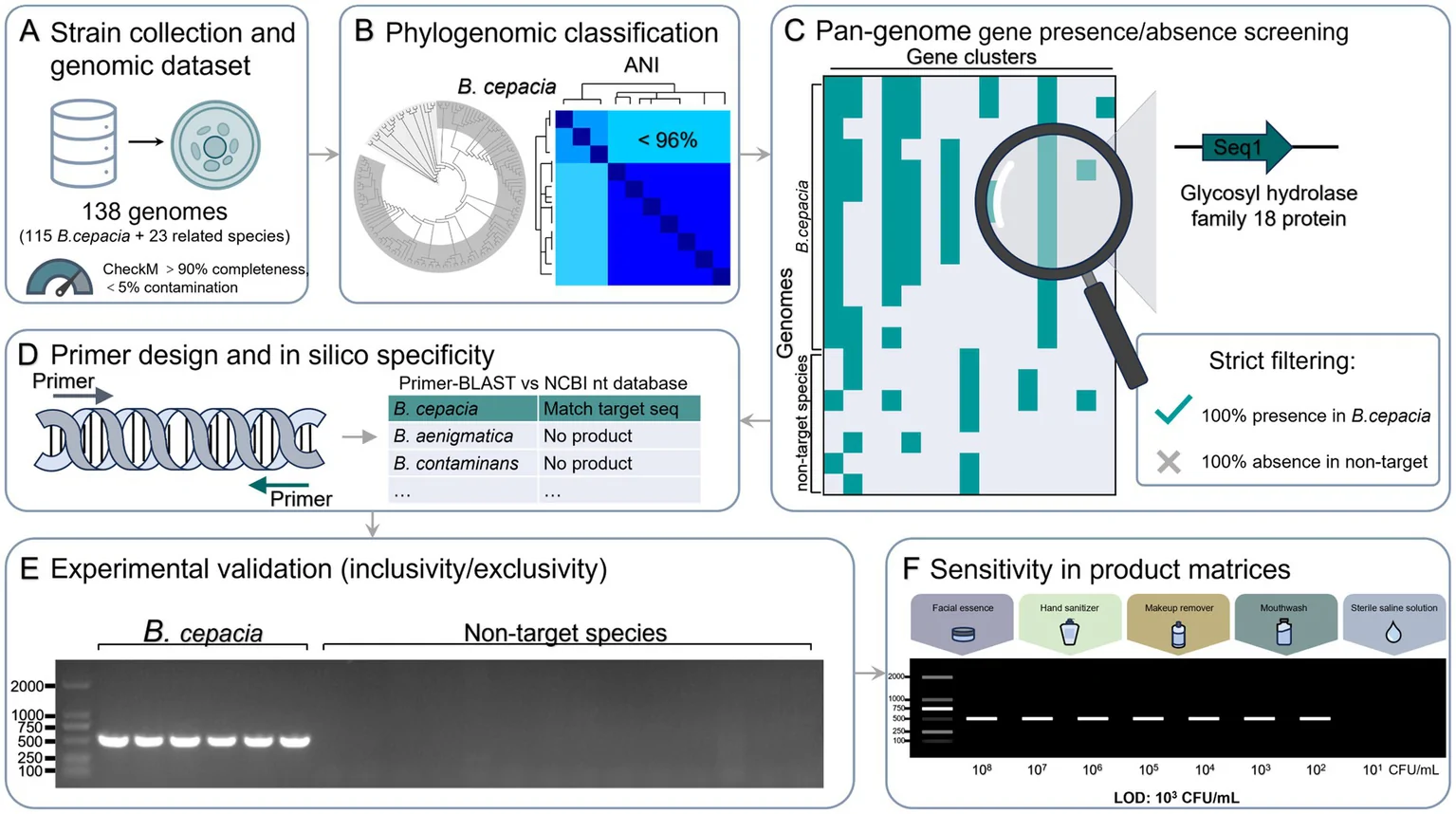
The mining process for species-specific molecular marker of *B. cepacia* and their practical application.

**Figure 3:**
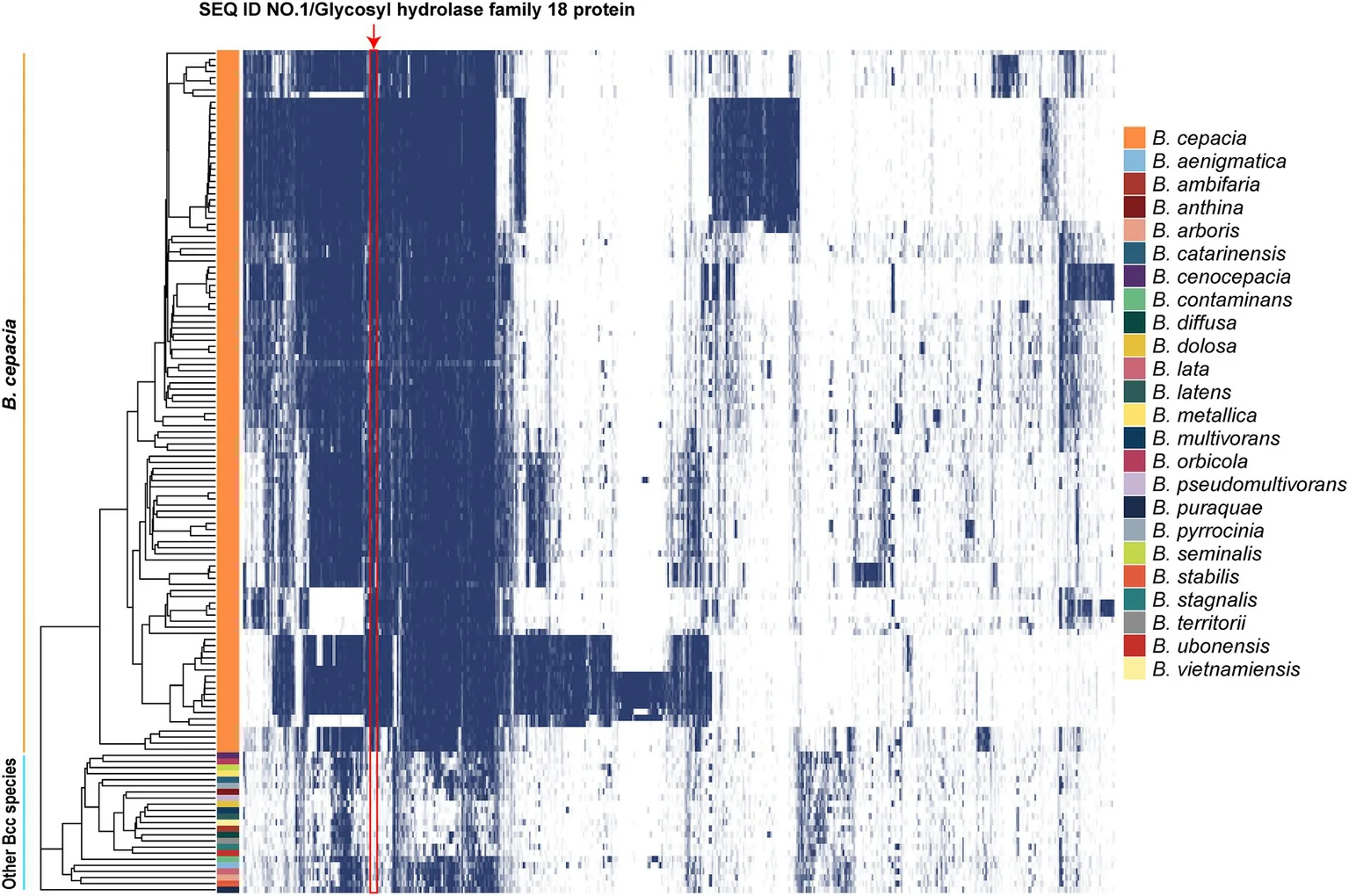
Gene present/absence in pan-genome analysis among strains of *B. cepacia* and other Bcc member species. The pan-genome comprises a total of 57,722 genes. Genes with distribution proportions ranging from 5 to 95% (10,834 genes) are displayed here. The region corresponding to the marker gene SEQ ID NO.1 (selected for subsequent species-specific primer design) is highlighted with a red box.

### 3.3 *In silico* specificity of the newly designed primer pairs

The specificity of the four candidate *B. cepacia* species-specific primer pairs was first assessed by *in silico* PCR using 283 *B. cepacia* genomes and 1,829 other Bcc genomes obtained from the NCBI database. Among these, the primer pair comprising SEQ ID NO.2 (5′-CTACGGTCAATAGCCAGTT-3′) and SEQ ID NO.3 (5′-GGATCGTCAAGCGGAATA-3′) demonstrated the best specificity performance, showing 100% inclusivity by generating amplicons of the expected size across all target genomes, and 100% exclusivity with no amplicon observed in non-target genomes (Supplementary Table S4). Based on these results, this primer pair was selected for further evaluation with experimental PCR assays.

### 3.4 Specificity and sensitivity of the primer pair with *in vitro* PCR assay

The specificity of the primer pair, comprising SEQ ID NO.2 and SEQ ID NO.3, was evaluated using a panel of reference strain pure cultures in this study (Supplementary Table S3). Gradient PCR optimization confirmed that an annealing temperature of 55 °C was optimal for this primer pair (Supplementary Figure S1). Inclusivity testing was performed with six target strains of *B. cepacia*, all of which yielded a single clear amplification band at the expected size of 529 bp (Figure 4A), confirming the high inclusivity of the primers and their suitability for accurate detection of *B. cepacia* strains. Conversely, exclusivity testing using 62 non-target strains belonging to diverse genera resulted in no amplification bands, demonstrating 100% exclusivity of the primer pair and the absence of nonspecific amplification under the PCR assay conditions (Figure 4A). Collectively, the above results indicate that the newly designed primer pair comprising SEQ ID NO.2 and SEQ ID NO.3, which targets the molecular gene marker SEQ ID NO.1, is highly specific and is capable of reliably identifying *B. cepacia* while distinguishing it from non-*B. cepacia* strains.

**Figure 4:**
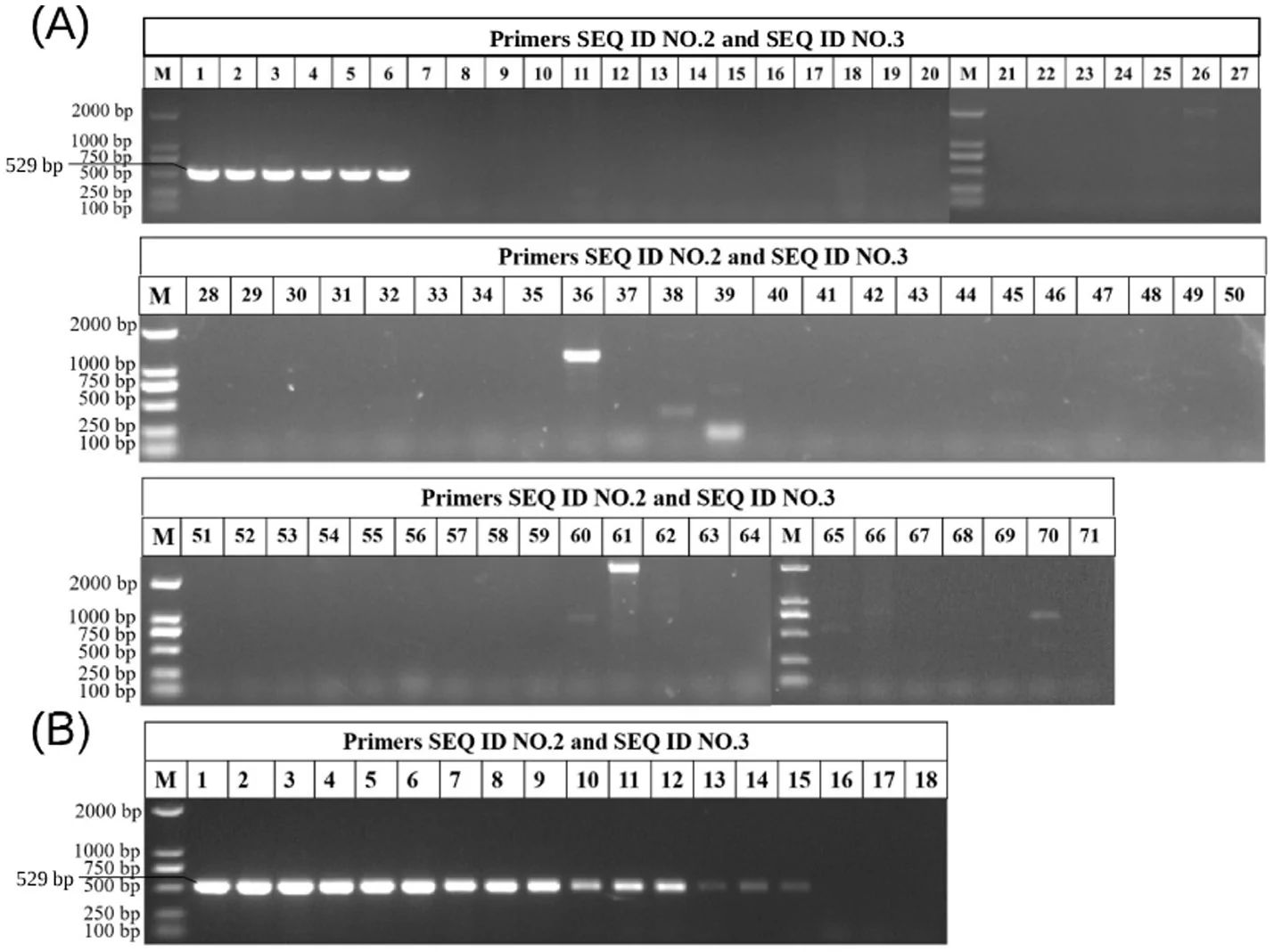
Specificity and sensitivity of the PCR assay targeting the *B. cepacia*-specific gene marker SEQ ID NO.1 using the primer pair of SEQ ID NO.2 and SEQ ID NO.3. **(A)** Inclusivity and exclusivity evaluation. Lanes 1–6: *B. cepacia* strains (inclusivity testing). Lanes 7–14 and 65–68: non-*B. cepacia* strains of Bcc species (exclusivity testing). Lanes 15–26, 28–63, and 69–70: non-target strains from diverse bacterial genera (exclusivity testing). Lanes 27 and 64: Blank control with no template. M: 2K DNA marker. The expected amplicon size is 529 bp. A detailed list of all strains tested, with corresponding lane numbers, is provided in Supplementary Table S3. **(B)** Sensitivity evaluation. Lanes 1–15: Triplicate PCR reactions at five DNA concentrations of *B. cepacia* strain CICC 21624^T^ genomic DNA (50, 5, 0.5, 0.05, and 0.005 ng/μL, respectively). 16–18: Blank control with no template. M: 2K DNA marker. The expected amplicon size is 529 bp. The detection limit was determined as 5 pg/μL.

The sensitivity of the primer pair was assessed via a serial 10-fold dilution of genomic DNA extracted from strain *B. cepacia* CICC 21624^T^. PCR amplification using SEQ ID NO.2 and SEQ ID NO.3 showed detection down to a DNA concentration of 5 pg/μL (Figure 4B). This low detection limit indicates that our developed PCR assay possesses high sensitivity for the detection of *B. cepacia* strains.

### 3.5 Evaluation of the sensitivity in water-based matrices with species-specific PCR

To evaluate the suitability of the PCR assay, genomic DNA was extracted from facial essence, hand sanitizer, makeup remover, mouthwash and sterile saline solution that had been artificially contaminated with different concentrations of a pure culture of *B. cepacia*. The experimental results of detection limits in different matrices were shown in Supplementary Figure S3; Table S7, and were visually presented in Figure 5. The PCR amplification results revealed that the sensitivity of the newly developed primer pair in all five artificially contaminated products was 10^3^ CFU/mL. These results demonstrated that the gene marker identified in this study has high sensitivity and can be utilized for the specific detection of *B. cepacia* in artificially spiked personal care and medical device products.

**Figure 5:**
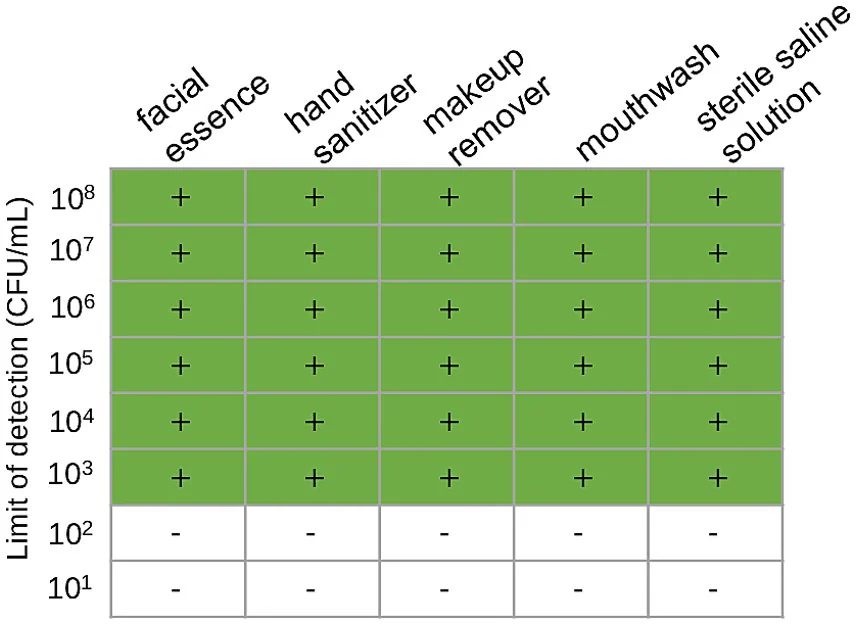
Detection limits of the assay in various sample matrices. Matrices spiked with different concentrations of *B. cepacia* pure culture were tested with the lowest concentration yielding a positive amplification signal recorded as the detection limit. “+” indicates a positive PCR result, while “−” indicates no detectable amplification.

## 4 Discussion

*B. cepacia* strains, members of Bcc, are ubiquitously distributed in the environment, colonizing diverse niches such as water and soil (Garrity et al., 2005). They exhibit remarkable resilience in hostile environments and have a propensity to form biofilms, which substantially reduces the efficacy of conventional disinfectants (Garrity et al., 2005; Tavares et al., 2020). Since the 1990s, Bcc, with *B. cepacia sensu stricto* as its representative member, has emerged as a major concern in the contamination of water-based products, leading to frequent products recalls (Jimenez, 2007; U.S. Food and Drug Administration, 2025). Consequently, Bcc is now considered an objectionable microorganism, attracting significant attention from industry stakeholders and regulatory authorities (Jimenez, 2007; U.S. Food and Drug Administration, 2025). Therefore, accurate identification of *B. cepacia sensu stricto* is critical for product quality control and contamination traceability. In the present study, we developed specific primers and employed conventional PCR to achieve rapid and convenient species-level identification of *B. cepacia*.

In early stages, the design of specific primers primarily relied on target sequences derived from experiments involving a limited number of strain cultures (La Duc et al., 2004; Naser et al., 2007). With the advent of high-throughput gene sequencing, a vast amount of genome data has accumulated in public databases, and numerous potential genes have been predicted and annotated (Wu et al., 2009; Mukherjee et al., 2017). This progress has enabled the mining of more precise and specific molecular markers based on genomic sequences. Currently, pan-genome analysis offers a novel strategy for the exploration of gene markers, increasingly applied to design more species-specific molecular markers even among closely related species (Kim, 2025). Unlike approaches that rely on single-genome, pan-genome analysis integrates multiple genomes from representative strains (Vernikos et al., 2015). By virtue of sequence alignment and phylogenetic tree construction, it can identify core genes that are unique to a particular species or strain. These genes serve as ideal candidate regions for the subsequent development of species- or strain- specific primers. However, it is important to note that the accuracy of pan-genome analysis can vary depending on the available genomic datasets (Kim, 2025). To generate distinguishing biomarkers as target regions for primer design, it is recommended that a sufficient and representative set of genome sequences must be incorporated into the analysis (Xiang et al., 2023). This set should include strains of the target species to ensure inclusivity, as well as closely related neighboring species to assess exclusivity. Such comprehensive inclusion is essential for obtaining reliable biomarkers. In addition, high-quality genomic data are critical for this purpose (Kim, 2025). Previous reports have indicated that insufficient genome integrity or incorrect taxonomic assignment may compromise the specificity of the selected molecular markers (Kim et al., 2022; Xiang et al., 2023), potentially leading to cross-reactions such as false positives or false negatives in practical applications. Therefore, genome sequences used in pan-genome analysis should undergo rigorous quality control and taxonomic verification. In this study, we developed specific primers for *B. cepacia* using a pan-genome approach based on 138 recruited genome sequences that covered both *B. cepacia* and its close relative species within Bcc. The sequences were qualified in terms of sequence quality, and their taxonomic positions were validated through ANI analysis to ensure reliability. Although PCR-based methods developed through pan-genome analysis have been applied in the detection of harmful microorganisms, such as *Salmonella*, *Cronobacter*, *Staphylococcus*, and *Listeria* (Vernikos et al., 2015; Kim, 2025), as well as beneficial microorganisms like lactic acid bacteria (Kim et al., 2022; Song et al., 2024), no such application has been reported for the identification of *B. cepacia*. Therefore, the procedure established in this study provides a valuable reference for mining species-level identification markers within Bcc using pan-genome analysis.

Primer specificity is a crucial determinant in PCR-based microbial identification methods. In this study, through pan-genomic analysis, we revealed that the gene encoding a glycosyl hydrolase family 18 (GH18) protein was highly conserved across all the 115 analyzed *B. cepacia* strains (sequence similarity: 90.3–100.0%; data not shown) but was completely absent from the 23 type strains of other Bcc species examined in this study. GH18 family proteins primarily exhibit chitinase activity, cleaving the β-1,4 glycosidic bonds in chitin, the second most abundant naturally synthesized polymer, to generate N-acetylglucosamine oligosaccharides, which can be utilized by bacteria as a nutrient source (Chen et al., 2020). *B. cepacia* has been reported to be widely distributed in soil, plant rhizospheres, and aquatic environments (Garrity et al., 2005), and a chitinase from *B. cepacia* strain KH2 was purified and characterized as early as 2002 (Ogawa et al., 2002). Thus the species-specific distribution pattern of the GH18-encoding gene provides a desirable molecular biomarker for the rapid identification of *B. cepacia*, as demonstrated by our *in silico* specificity analysis and experimental validation.

Systematic validation is a prerequisite for the practical application of any new method. Several standards and documents provide practical guidelines for method validation, including the ISO 16140 series (ISO 16140-6, 2019; ISO 16140-7, 2024), ISO 22118 (ISO 22118, 2011), ICH Q2(R1) *Validation of Analytical Procedures Text and Methodology* (ICH Q2(R1), 2005) and USP < 1,223 > *Validation of Alternative Microbiological Methods* (USP, 2015). By adapting selected performance criteria and concepts from these guidelines, we validated the specificity of the newly designed primer pair for *B. cepacia* species using various reference strains and genome sequences (Figure 4A; Supplementary Tables S3, S4). The results demonstrated high inclusivity and exclusivity of the primer pair for *B. cepacia*. Consequently, the PCR method based on this specific primer pair offers a convenient and cost-effective tool for the detection of *B. cepacia* in personal care or medical device matrices. For microbial pure culture, the sensitivity of the PCR assay for *B. cepacia* was determined to be 5 pg/μL of genomic DNA, meaning that each PCR reaction can detect *B. cepacia* from as little as 0.01 ng of DNA. This detection limit is lower than the quantity of template genomic DNA recommended for conventional PCR reactions (Dieffenbach and Dveksler, 2003). For artificially contaminated products, the sensitivity of the assay was 10^3^ CFU/mL for all five water-based matrices (Figure 5; Supplementary Table S7; Figure S3), including facial essence, hand sanitizer, makeup remover, mouthwash and sterile saline solution. This level of sensitivity is comparable to those reported for some other bacterial species in various sample matrices, such as lactic acid bacteria from cold-smoked salmon (10^3^ CFU/g) and *Lacticaseibacillus paracasei* in probiotic products (10^3^ CFU/mL) (Guo et al., 2024), indicating that our PCR method using the species-specific primer pair is highly sensitive. Notably, personal care and medical device products typically contain complex ingredients (e.g., oils, polymers, preservatives, and suspended particles) that may interfere with nucleic acid extraction or thereby inhibit PCR amplification (Bermond et al., 2022). Our inhibition control experiments in which positive DNA was spiked into all negative samples showed that no detectable PCR inhibition occurred, thereby ensuring the reliability of the detection results in these complex matrices.

The detection method for *B. cepacia* established in this study relies on the specificity of the designed primer pair. Although a number of genome sequences were used to validate the primer pair, only a limited number of bacterial pure cultures were tested. No clinical or environmental *B. cepacia* isolates beyond the CICC collection were tested and strains of *B. stabilis*, *B. pyrrocinia*, and *B. ambifaria*, known to be phenotypically and genetically close to *B. cepacia*, have not been included in the panel. Therefore, more new isolates are needed to further confirm the evolutionary stability of the targeted biomarker. Additionally, this method cannot distinguish between dead and viable cells; the incorporation of viability dyes such as propidium monoazide is required for the detection of viable cells. In future studies, we aim to extend the application of this research paradigm to other species within Bcc or to other difficult-to-identify microbial groups. This would facilitate the discovery of novel molecular targets and the establishment of rapid molecular detection methods for preliminary contamination screening, thereby helping to prevent public health incidents caused by substandard products.

## 5 Conclusion

In conclusion, we developed a novel PCR assay for species-specific detection of *B. cepacia* based on a conserved GH18 gene marker identified through pan-genome analysis of 138 genome sequences. The method was validated with both *in silico* specificity analysis and *in vitro* PCR assay, demonstrating high inclusivity, exclusivity, and sensitivity (5 pg/μL DNA; 10^3^ CFU/mL in contaminated water-based personal care and medical device products). This convenient and cost-effective tool supports preliminary contamination screening for personal care or medical device products, and may be adapted to other product categories, including foods, following appropriate matrix-specific validation. To our knowledge, this is the first application of pan-genome analysis for species-level marker discovery within Bcc. The research paradigm can be extended to other Bcc species and closely related microbial groups that are difficult to resolve taxonomically, facilitating the development of rapid molecular detection methods to enhance product safety and reduce public health risks associated with substandard products.

## Data Availability

The datasets presented in this study can be found in online repositories. The names of the repository/repositories and accession number(s) can be found in the article/Supplementary material.

## References

[ref1] Addou S. Rentzsch R. Lee D. Orengo C. A. (2009). Domain-based and family-specific sequence identity thresholds increase the levels of reliable protein function transfer. J. Mol. Biol. 387, 416–430. doi: 10.1016/j.jmb.2008.12.045, 19135455

[ref2] Aytaç Ö. Tanrıverdi E. S. Gündağ Ö. Şenol F. F. Karlıdağ G. E. Otlu B. (2025). An intensive care outbreak caused by *Burkholderia cepacia* from bacterial filters. Pathogens 14:266. doi: 10.3390/pathogens14030266, 40137751 PMC11945276

[ref3] Bermond C. Cherrad S. Trainoy A. Ngari C. Poulet V. (2022). Real-time qPCR to evaluate bacterial contamination of cosmetic cream and the efficiency of protective ingredients. J. Appl. Microbiol. 132, 2106–2120. doi: 10.1111/jam.15310, 34586708

[ref4] Burkholder W. H. J. P. (1950). Sour skin, a bacterial rot of onion bulbs. Phytopathology 40, 115–117.

[ref5] Chen W. Jiang X. Yang Q. (2020). Glycoside hydrolase family 18 chitinases: the known and the unknown. Biotechnol. Adv. 43:107553. doi: 10.1016/j.biotechadv.2020.107553, 32439576

[ref6] Coulon P. M. L. Zlosnik J. E. A. Déziel E. (2021). Presence of the Hmq system and production of 4-hydroxy-3-methyl-2-alkylquinolines are heterogeneously distributed between *Burkholderia cepacia* complex species and more prevalent among environmental than clinical isolates. Microbiol. Spectrum 9:e0012721. doi: 10.1128/Spectrum.00127-21, 34132614 PMC8552760

[ref7] Delcher A. L. Bratke K. A. Powers E. C. Salzberg S. L. (2007). Identifying bacterial genes and endosymbiont DNA with glimmer. Bioinformatics 23, 673–679. doi: 10.1093/bioinformatics/btm009, 17237039 PMC2387122

[ref8] Devanga Ragupathi N. K. Veeraraghavan B. (2019). Accurate identification and epidemiological characterization of *Burkholderia cepacia* complex: an update. Ann. Clin. Microbiol. Antimicrob. 18:7. doi: 10.1186/s12941-019-0306-0, 30717798 PMC6360774

[ref9] Dieffenbach C. W. Dveksler G. S. (2003). PCR Primer: A Laboratory Manual. New York: Cold Spring Harbor Laboratory Press.

[ref10] Doran J. Foster C. Saunders M. Chandra N. L. Turton J. F. Kenna D. T. . (2025). Two concurrent nationwide healthcare-associated outbreaks of *Burkholderia cepacia* complex linked to product contamination, UK and Ireland, 2010-2023. Infect. Control Hosp. Epidemiol. 46, 1006–1012. doi: 10.1017/ice.2025.10232, 40905274

[ref11] European Food Safety Authority (2024). EFSA statement on the requirements for whole genome sequence analysis of microorganisms intentionally used in the food chain. EFSA J. 22:e8912. doi: 10.2903/j.efsa.2024.8912, 39135845 PMC11317806

[ref12] Fu L. Niu B. Zhu Z. Wu S. Li W. (2012). CD-HIT: accelerated for clustering the next-generation sequencing data. Bioinformatics 28, 3150–3152. doi: 10.1093/bioinformatics/bts565, 23060610 PMC3516142

[ref13] Furlan J. P. R. Pitondo-Silva A. Braz V. S. Gallo I. F. L. Stehling E. G. (2019). Evaluation of different molecular and phenotypic methods for identification of environmental *Burkholderia cepacia* complex. World J. Microbiol. Biotechnol. 35:39. doi: 10.1007/s11274-019-2614-0, 30739255

[ref14] Garrity G. Brenner D. Kreig N. Staley J. (2005). Bergey’s Manual of Systematic Bacteriology Vol. 2 Part C. The *Alpha*-, *Beta*-, *Delta*-, and *Epsilonproteobacteria*. New York: Springer.

[ref15] Guo L. Ze X. Jiao Y. Song C. Zhao X. Song Z. . (2024). Development and validation of a PMA-qPCR method for accurate quantification of viable *Lacticaseibacillus paracasei* in probiotics. Front. Microbiol. 15:1456274. doi: 10.3389/fmicb.2024.1456274, 39171269 PMC11335531

[ref16] ICH Q2(R1) (2005). Validation of analytical procedures: text and methodology. Available online at: https://www.fda.gov/media/152208/download (Accessed May 13, 2006).

[ref17] ISO 16140-6 (2019). ISO 16140-6:2019 microbiology of the food chain — method validation part 6: protocol for the validation of alternative (proprietary) methods for microbiological confirmation and typing procedures. Available online at: https://www.iso.org/obp/ui/en/#iso:std:iso:16140:-6:ed-1:v1:en

[ref18] ISO 16140-7 (2024). ISO 16140-7:2024 microbiology of the food chain — method validation part 7: protocol for the validation of identification methods of microorganisms. Available online at: https://www.iso.org/obp/ui/en/#iso:std:iso:16140:-7:ed-1:v1:en

[ref19] ISO 22118 (2011). ISO 22118:2011 microbiology of food and animal feeding stuffs — polymerase chain reaction (PCR) for the detection and quantification of food-borne pathogens — performance characteristics. Available online at: https://www.iso.org/obp/ui/en/#iso:std:iso:22118:ed-1:v1:en

[ref20] Jimenez L. (2007). Microbial diversity in pharmaceutical product recalls and environments. PDA J. Pharm. Sci. Technol. 61, 383–399. 18047177

[ref21] Jin Y. Zhou J. Zhou J. Hu M. Zhang Q. Kong N. . (2020). Genome-based classification of *Burkholderia cepacia* complex provides new insight into its taxonomic status. Biol. Direct. 15:6. doi: 10.1186/s13062-020-0258-5, 32131884 PMC7057466

[ref22] Kim S. S. (2025). Application of comparative genomics for the development of PCR primers for the detection of harmful or beneficial microorganisms in food: mini-review. Foods 14:1060. doi: 10.3390/foods14061060, 40232097 PMC11942385

[ref23] Kim Y. Gu C. Kim H. U. Lee S. Y. (2020). Current status of pan-genome analysis for pathogenic bacteria. Curr. Opin. Biotechnol. 63, 54–62. doi: 10.1016/j.copbio.2019.12.001, 31891864

[ref24] Kim E. Yang S. M. Kim I. S. Lee S. Y. Kim H. Y. (2022). Identification of *Leuconostoc* species based on novel marker genes identified using real-time PCR via computational pangenome analysis. Front. Microbiol. 13:1014872. doi: 10.3389/fmicb.2022.1014872, 36212836 PMC9537375

[ref25] La Duc M. T. Satomi M. Agata N. Venkateswaran K. (2004). *gyrB* as a phylogenetic discriminator for members of the *Bacillus anthracis*-*cereus*-*thuringiensis* group. J. Microbiol. Methods 56, 383–394. doi: 10.1016/j.mimet.2003.11.004, 14967230

[ref26] Lee I. Ouk Kim Y. Park S. C. Chun J. (2016). OrthoANI: an improved algorithm and software for calculating average nucleotide identity. Int. J. Syst. Evol. Microbiol. 66, 1100–1103. doi: 10.1099/ijsem.0.000760, 26585518

[ref27] Letunic I. Bork P. (2024). Interactive tree of life (iTOL) v6: recent updates to the phylogenetic tree display and annotation tool. Nucleic Acids Res. 52, W78–w82. doi: 10.1093/nar/gkae268, 38613393 PMC11223838

[ref28] Mahenthiralingam E. Urban T. A. Goldberg J. B. (2005). The multifarious, multireplicon *Burkholderia cepacia* complex. Nat. Rev. Microbiol. 3, 144–156. doi: 10.1038/nrmicro1085, 15643431

[ref29] Moore J. E. McIlhatton B. Shaw A. Murphy P. G. Elborn J. S. (2001). Occurrence of *Burkholderia cepacia* in foods and waters: clinical implications for patients with cystic fibrosis. J. Food Prot. 64, 1076–1078. doi: 10.4315/0362-028X-64.7.1076, 11456196

[ref30] Mukherjee S. Seshadri R. Varghese N. J. Eloe-Fadrosh E. A. Meier-Kolthoff J. P. Göker M. . (2017). 1,003 reference genomes of bacterial and archaeal isolates expand coverage of the tree of life. Nat. Biotechnol. 35, 676–683. doi: 10.1038/nbt.3886, 28604660

[ref31] Naser S. M. Dawyndt P. Hoste B. Gevers D. Vandemeulebroecke K. Cleenwerck I. . (2007). Identification of lactobacilli by *pheS* and *rpoA* gene sequence analyses. Int. J. Syst. Evol. Microbiol. 57, 2777–2789. doi: 10.1099/ijs.0.64711-0 18048724

[ref32] Ogawa K. Yoshida N. Kariya K. Ohnishi C. Ikeda R. (2002). Purification and characterization of a novel chitinase from *Burkholderia cepacia* strain KH2 isolated from the bed log of *Lentinus edodes*, shiitake mushroom. J. Gen. Appl. Microbiol. 48, 25–33. doi: 10.2323/jgam.48.25 12469313

[ref33] Palleroni N. J. Holmes B. (1981). *Pseudomonas cepacia* sp. nov., nom. rev. 31, 479–481. doi: 10.1099/00207713-31-4-479

[ref34] Parks D. H. Imelfort M. Skennerton C. T. Hugenholtz P. Tyson G. W. (2015). CheckM: assessing the quality of microbial genomes recovered from isolates, single cells, and metagenomes. Genome Res. 25, 1043–1055. doi: 10.1101/gr.186072.114, 25977477 PMC4484387

[ref35] Quiroz-Morales S. E. Muriel-Millán L. F. Ponce-Soto G. Y. González-Valdez A. Castillo-Juárez I. Servín-González L. . (2023). *Pseudomonas aeruginosa* strains belonging to phylogroup 3 frequently exhibit an atypical quorum sensing response: the case of MAZ105, a tomato rhizosphere isolate. Microbiology (Reading) 169:001401. doi: 10.1099/mic.0.001401, 37819040 PMC10634362

[ref36] Richter M. Rosselló-Móra R. (2009). Shifting the genomic gold standard for the prokaryotic species definition. Proc. Natl. Acad. Sci. 106, 19126–19131. doi: 10.1073/pnas.0906412106, 19855009 PMC2776425

[ref37] Riesco R. Trujillo M. E. (2024). Update on the proposed minimal standards for the use of genome data for the taxonomy of prokaryotes. Int. J. Syst. Evol. Microbiol. 74:006300. doi: 10.1099/ijsem.0.006300, 38512750 PMC10963913

[ref38] Seelman S. L. Bazaco M. C. Wellman A. Hardy C. Fatica M. K. Huang M. J. . (2022). *Burkholderia cepacia* complex outbreak linked to a no-rinse cleansing foam product, United States – 2017–2018. Epidemiol. Infect. 150:e154. doi: 10.1017/s0950268822000668, 35923078 PMC9428903

[ref39] Singh V. K. Mangalam A. K. Dwivedi S. Naik S. (1998). Primer premier: program for design of degenerate primers from a protein sequence. BioTechniques 24, 318–319. doi: 10.2144/98242pf02, 9494736

[ref40] Song Z. Ge Y. Yu X. Liu R. Liu C. Cheng K. . (2024). Development of a single nucleotide polymorphism-based strain-identified method for *Streptococcus thermophilus* CICC 6038 and *Lactobacillus delbrueckii* ssp. *bulgaricus* CICC 6047 using pan-genomics analysis. J. Dairy Sci. 107, 4248–4258. doi: 10.3168/jds.2023-23655, 38246550

[ref41] Spilker T. Baldwin A. Bumford A. Dowson C. G. Mahenthiralingam E. LiPuma J. J. (2009). Expanded multilocus sequence typing for *Burkholderia* species. J. Clin. Microbiol. 47, 2607–2610. doi: 10.1128/jcm.00770-09, 19494070 PMC2725695

[ref42] Tavares M. Kozak M. Balola A. Sá-Correia I. (2020). *Burkholderia cepacia* complex bacteria: a feared contamination risk in water-based pharmaceutical products. Clin. Microbiol. Rev. 33:e00139-19. doi: 10.1128/cmr.00139-19, 32295766 PMC7194853

[ref43] U.S. Food and Drug Administration (2025). Microbiological Quality Considerations in non-sterile Drug Manufacturing: Guidance for Industry. Silver Spring, MD: U.S. Department of Health and Human Services, Food and Drug Administration, Center for Drug Evaluation and Research (CDER).

[ref44] USP (2015). USP General Chapter <1223> Validation of alternative microbiological methods. Available online at: https://doi.usp.org/USPNF/USPNF_M99943_03_01.html

[ref45] Vernikos G. Medini D. Riley D. R. Tettelin H. (2015). Ten years of pan-genome analyses. Curr. Opin. Microbiol. 23, 148–154. doi: 10.1016/j.mib.2014.11.016, 25483351

[ref46] Vilella A. J. Severin J. Ureta-Vidal A. Heng L. Durbin R. Birney E. (2009). EnsemblCompara GeneTrees: complete, duplication-aware phylogenetic trees in vertebrates. Genome Res. 19, 327–335. doi: 10.1101/gr.073585.107, 19029536 PMC2652215

[ref47] Wu D. Hugenholtz P. Mavromatis K. Pukall R. Dalin E. Ivanova N. N. . (2009). A phylogeny-driven genomic encyclopaedia of Bacteria and Archaea. Nature 462, 1056–1060. doi: 10.1038/nature08656, 20033048 PMC3073058

[ref48] Xiang X. Lu J. Xu X. Hou X. Diao E. Qian S. . (2023). Rapid identification of novel specific molecular targets for PCR detection of four *Enterococcus* species. LWT-Food Sci. Technol. 173:114356. doi: 10.1016/j.lwt.2022.114356

[ref49] Yabuuchi E. Kosako Y. Oyaizu H. Yano I. Hotta H. Hashimoto Y. . (1992). Proposal of *Burkholderia* gen. nov. and transfer of seven species of the genus *Pseudomonas* homology group II to the new genus, with the type species *Burkholderia cepacia* (Palleroni and Holmes 1981) comb. nov. Microbiol. Immunol. 36, 1251–1275. doi: 10.1111/j.1348-0421.1992.tb02129.x, 1283774

