# Supplementary material for: Pan-genome analysis identifies a Burkholderia cepacia-specific gene marker for rapid species-level identification
Source: Front Microbiol. 2026 Sep 23;17:1915686. doi: 10.3389/fmicb.2026.1915686 (PMC13642291; doi:10.3389/fmicb.2026.1915686)
Supplement: Supplementary file 1 [file Data_Sheet_1.docx]

**Supplementary Figure S1.** Gradient PCR optimization for the primer pair comprising of SEQ ID NO.2 and SEQ ID NO.3. PCR products were amplified from genomic DNA of *B. cepacia* CICC 21624ᵀ using annealing temperatures ranging from 47°C to 59°C in 2°C increments (lanes 1–7: 47, 49, 51, 53, 55, 57 and 59°C, respectively). Lane 8, blank control (no template DNA). M, DNA molecular weight marker (with sizes indicated in bp). Expected amplicon: 529 bp. All tested annealing temperatures yielded detectable specific amplification products. To achieve optimal specificity, the relatively higher annealing temperature of 55°C (lane 5) was selected for subsequent experiments.

**Supplementary Figure S2.** Heatmap of ANI values among 24 type strains of Bcc species.

**Supplementary Figure S3**. PCR detection limits in five spiked matrices. Agarose gel images showing amplification of *B. cepacia* in (A) sterile saline solution, (B) facial essence, (C) hand sanitizer, (D) mouthwash, and (E) makeup remover spiked with serial 10-fold dilutions of *B. cepacia* strain ATCC 25416^T^ (10⁸ to 10¹ CFU/mL). For each matrix, lanes 1-24 represent triplicate PCR reactions at eight concentrations (10⁸ to 10¹ CFU/mL, three replicates per concentration). Lanes 25-30 represent inhibition controls: triplicate PCR reactions at 10^2^ and 10¹ CFU/mL (the concentrations below the LOD), in which purified genomic DNA from *B. cepacia* strain ATCC 25416^T^ was spiked into each negative PCR reaction to verify the absence of PCR inhibitors in the extracted DNA. Red boxes indicate the LOD at 10^3^ CFU/mL (lanes 16-18). M, DNA marker. Expected amplicon: 529 bp.
